# Disseminated Nocardia Brain Abscess Presenting as Primary Lung Cancer With Brain Metastasis

**DOI:** 10.7759/cureus.43631

**Published:** 2023-08-17

**Authors:** Swechchha Silwal, Mikael Mir, Sydney Boike, Karuna Bista, Sumeet K Yadav, Jessica Sheehy, Syed Anjum Khan, Eric O Gomez Urena

**Affiliations:** 1 Internal Medicine, Trinity Health Oakland/Wayne State University, Pontiac, USA; 2 Medicine, University of Minnesota Medical School, Minneapolis, USA; 3 Internal Medicine, Nepal Medical College, Kathmandu, NPL; 4 Internal Medicine, Mayo Clinic Health System, Mankato, USA; 5 Infectious Diseases, Mayo Clinic Health System, Mankato, USA; 6 Critical Care Medicine, Mayo Clinic Health System, Mankato, USA

**Keywords:** nocardia, nocardia spp, skin abscess, nocardia brasiliensis, fungal brain abscess, nocardia brain infection

## Abstract

We present a challenging case of disseminated *Nocardia brasiliensis *infection manifesting as brain and skin abscesses. *Nocardia* is an important potential pathogen to consider in patients with a relevant travel history to endemic regions or atypical presentations, such as brain and skin abscesses. About one-third of patients with *Nocardia* infections are immunocompetent, and their symptoms are nonspecific. This case shows the limitations of imaging studies in diagnosing *Nocardia* brain abscesses, as the patient’s non-magnetic resonance (MR) conditional pacemaker precluded MRI evaluation and led to a diagnostic challenge. Therefore, the patient’s initial evaluation was presumed to be primary lung cancer with brain metastasis. High clinical suspicion, imaging studies (especially MRI), and tissue biopsy are needed to diagnose this type of brain abscess in a timely manner to prevent further complications.

## Introduction

*Nocardia* species are aerobic gram-positive bacteria that are classified as aerobic actinomycetes. More than 90 species have been described, and 54 species have been reported to infect humans [1,2]. They are found in soil, water, and decaying vegetation and can transmit to humans through inhalation or direct skin contact with contaminated material. *Nocardia* usually causes infection in patients with immunosuppressive conditions, such as those with HIV/AIDS, organ transplants, or long-term corticosteroid use, but one-third of patients are immunocompetent [3,4]. In immunocompetent patients, symptoms are nonspecific, and pulmonary nocardiosis can present with fever, cough, and unintentional weight loss [5]. Brain abscesses in patients with *Nocardia* are rare. They can present with headaches, fever, neurological deficits, seizures, and change in mental status. Diagnosing a brain abscess will require a combination of high clinical suspicion, imaging studies (especially MRI), and tissue biopsy to prove the infection [6]. Here, we present an interesting case of *Nocardia* brain abscess presenting as presumed primary lung cancer with brain metastasis.

## Case presentation

The patient is a 72-year-old female with a past medical history significant for diabetes mellitus, hypertension, sinus node dysfunction status post pacemaker, right hemispheric stroke, and atrial fibrillation on anticoagulation therapy and with a recent travel history to Arizona, who presented to the hospital for the evaluation of aphasia and worsening right-sided weakness.

**Figure 1:**
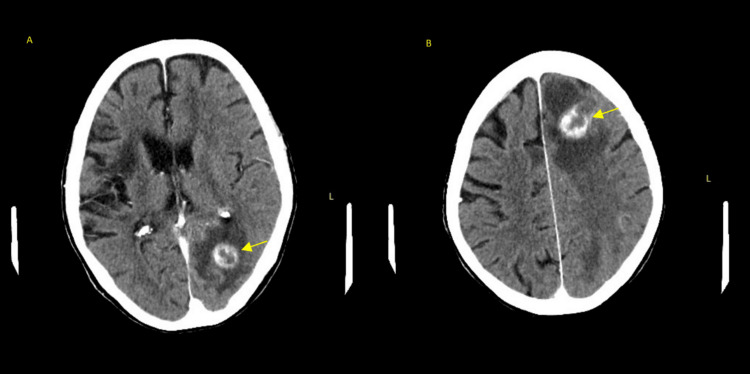
Mass-like enhancing lesion in the left occipital (A) and left frontal lobe (B) with vasogenic edema.

**Figure 2:**
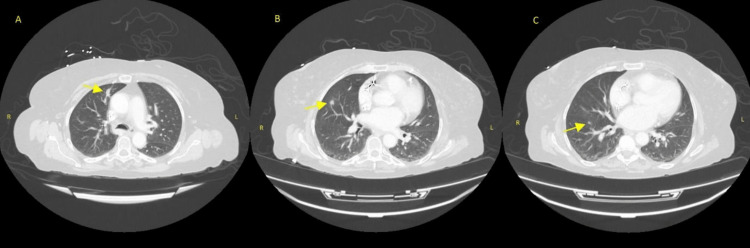
(A) An 18 × 7 mm anterior right upper lobe nodule, (B) an 8 × 7 mm nodule right lung base, and (C) a 5 mm nodule right lung base.

A CT scan of the head was obtained and showed a prominent area of hypoattenuation in the left frontal and occipital lobe with vasogenic edema and associated mass effect. Per radiology, the appearance was most concerning for a neoplastic process such as metastasis; however, the changes could be related to an acute/subacute infarct. Brain MRI was recommended, but unfortunately, the patient had a non-magnetic resonance (MR) conditional pacemaker generator at the time, so the MRI could not be completed. The patient underwent a CT of the head with contrast and a CT of the chest, abdomen, and pelvis with contrast to evaluate for possible primary malignancy. Significant pulmonary nodules were noted concerning for possible primary lung cancer with metastatic brain lesions (Figures 1, 2). The patient was started on dexamethasone and levetiracetam for seizure prophylaxis.

A CT biopsy of the lung lesion was planned as an outpatient; however, before obtaining the biopsy, the patient developed worsening chest pain. She then returned to the emergency department and had a repeat chest CT, which showed multiple new round opacities. Metastatic disease was felt to be less likely, given the rapid evolution of symptoms. She also had a repeat CT of the head because of progressive weakness with aphasia, which was notable for an increase in the size of rim-enhancing masses with increasing surrounding edema. There was concern for a brain abscess given the rapid progression and the fact that the patient was on high-dose steroid therapy, so vasogenic edema was felt to be less likely.

**Figure 3:**
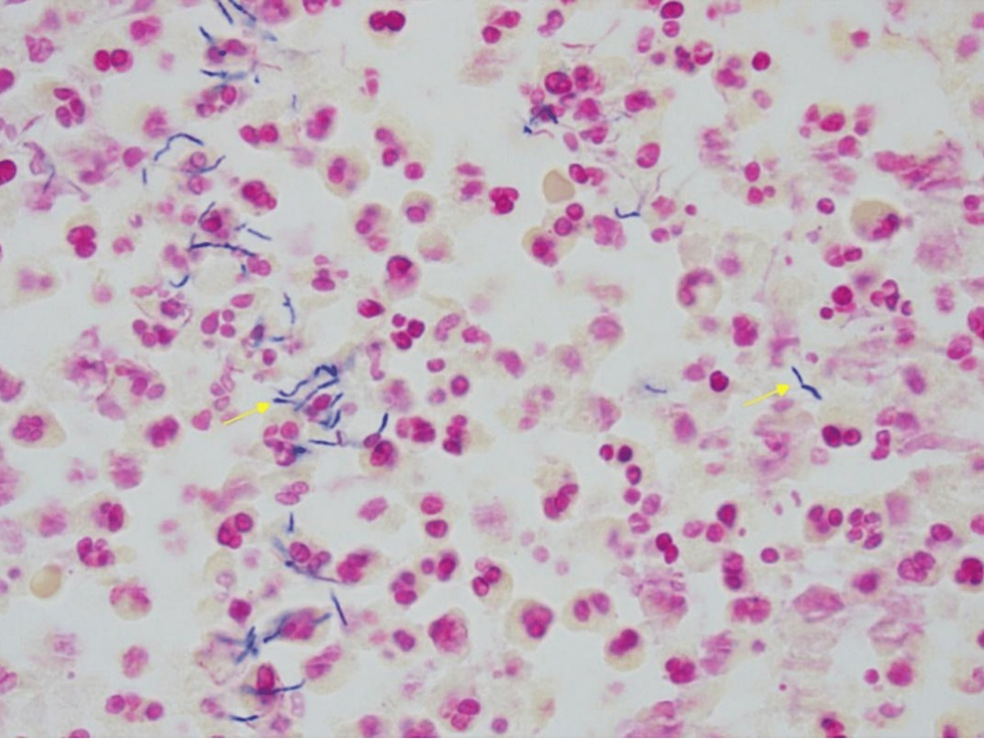
Gram stain showing branching bacillus consistent with Nocardia.

She was initially started on broad-spectrum antibiotics. Based on the distribution of lesions and the patient’s recent travel to Arizona, there was a concern for possible *Nocardia* infection versus endemic mycoses. Therefore, empiric antifungal therapy was added. The patient then underwent a CT-guided biopsy of the lung nodules, and the pathology report showed acute inflammation consistent with pneumonia and no evidence of neoplasm. Acid-fast bacilli (AFB) and Grocott’s methenamine silver (GMS) stains were negative. The patient also developed a boil while in Arizona that involved the right lateral thigh, which was painful. Ultrasound confirmed that this was an abscess, and the patient subsequently underwent incision and drainage of the area. The lung biopsy and skin abscess both grew *Nocardia brasiliensis*. The patient was able to ultimately undergo image-guided left frontal craniotomy and drainage of left frontal mass. Pathology showed branching bacillus consistent with *Nocardia* (Figure 3). Hence, the diagnosis of disseminated nocardiosis with cutaneous, pulmonary, and cerebral involvement was confirmed.

Trimethoprim-sulfamethoxazole and imipenem were started for disseminated nocardiosis. Her course was complicated by an acute kidney injury with thrombocytopenia. Antibiotic therapy was modified to ceftriaxone and minocycline. Susceptibility results then showed that the isolate had intermediate sensitivity to minocycline and resistance to ceftriaxone. This was followed by restarting imipenem and moxifloxacin for four weeks postoperatively. Eventually, she was able to be transitioned to trimethoprim-sulfamethoxazole and moxifloxacin to complete antibiotic therapy. Six months into treatment, the patient had a repeat chest CT that showed the complete resolution of the cavitary mass-like opacities and small nodules. A CT of the head showed resolving abscesses in the left frontal and occipital lobes with minimal residual findings. One year into treatment, an MRI compatible with her automated implantable cardioverter defibrillator (AICD) revealed no residual brain mass, and antibiotics were discontinued.

## Discussion

*Nocardia brasiliensis* is found primarily in the soil. It has been noted that *N. brasiliensis* is the most common cause of infection with *Nocardia* in immunocompetent hosts within the tropical and subtropical areas of the southern United States [2]. The risk factors for *Nocardia* infection include malignancy, HIV infection, long-term treatment with steroids, or suppressed cell-mediated immunity. Concurrent infections, cytomegalovirus infection, chronic obstructive pulmonary disease (COPD), diabetes mellitus, and alcoholism also have been associated with *Nocardia* infections. Pulmonary involvement is the most common clinical presentation of *Nocardia*, but it can also involve the skin and central nervous system (CNS) [7]. Pulmonary nocardiosis is usually acquired through inhalation or hematogenous dissemination, and primary cutaneous and soft tissue nocardiosis is usually from traumatic skin injury involving soil contamination. Extrapulmonary nocardiosis can occur through hematogenous dissemination or via contiguous spread of necrotizing pneumonitis or abscess [8,9]. The central nervous system, although rare, remains the most common extrapulmonary location for nocardiosis. *Nocardia* infection accounts for approximately 2% of all brain abscesses but increases the mortality risk by 30% compared to other causes. Brain abscesses secondary to *Nocardia* species are most commonly caused by *Nocardia farcinica*, which is responsible for over 80% of all cases of *Nocardia* brain abscess [4]. *Nocardia brasiliensis*, however, is most commonly implicated in skin infections [9].

Clinically, patients with pulmonary involvement present with fever, cough, and fatigue. CNS involvement leads to neurological symptoms, which develop gradually but can progress rapidly. Patients may also have a nonspecific clinical presentation, making diagnosing *Nocardia* more difficult. The differential diagnosis for pulmonary nocardiosis includes malignancy, mycobacterium infection, aspergillosis, and fungal infection [10]. Our patient had pulmonary involvement and extrapulmonary manifestations with brain and skin abscesses. She did not have any pulmonary symptoms on presentation and presented to the hospital secondary to neurological symptoms. Her initial working diagnosis was that her symptoms were related to a pulmonary malignancy with brain metastases. However, only with the rapid progression of disease symptoms, *Nocardia* became our primary differential. Unfortunately, during her initial workup, an MRI could not be obtained secondary to her pacemaker device, and this could have shown a ring-enhancing lesion, which would raise the clinical index of suspicion for infection [11]. The pathogenic and virulence factors of *N. brasiliensis* are not entirely understood, but it is thought to be related to the organism’s ability to evade phagocytosis [8]. Additionally, *Nocardia* exhibits varying degrees of acid-fastness, making it challenging to identify. Acid-fastness depends on the mycolic acid composition in the cell wall and the type of stain used. Modified acid-fast stains can help retain the color and aid in the diagnosis. In addition, *Nocardia *is slow-growing and may take up to two to three weeks to produce sufficient colonies for identification, which can contribute to diagnostic uncertainty [2]. If *Nocardia* is suspected in a patient, the laboratory should be informed to set the sample up for an extended period of incubation for identification. Newer techniques with gene sequencing, molecular techniques with polymerase chain reaction (PCR), and metagenomic next-generation sequencing may be available in specific laboratories to aid in an early diagnosis [12]. In our patient, an extended culture from the pulmonary biopsy and thigh abscess was requested, which confirmed the diagnosis of *Nocardia*.

*Nocardia* has variable antimicrobial susceptibility; therefore, management must be individualized based on the culture susceptibility results [13]. Sulfonamides, including sulfadiazine, sulfisoxazole, and trimethoprim-sulfamethoxazole, have remained the first choice of antimicrobial therapy. Alternatively, amikacin, imipenem, meropenem, ceftriaxone, cefotaxime, minocycline, moxifloxacin, levofloxacin, linezolid, tigecycline, and amoxicillin-clavulanic acid can be used [10]. However, some species of *Nocardia* tend to have higher degrees of multidrug resistance. Such species are *N. farcinica*, *N. brasiliensis*, and *N. otitidiscaviarum*. Therefore, combination therapy should be used for initial treatment until culture sensitivity is available [13]. Once there is clinical improvement, treatment with a single agent is appropriate, especially in cutaneous manifestations of *Nocardia*. Drugs with CNS penetration, such as trimethoprim-sulfamethoxazole and ceftriaxone, should be used in patients with CNS disease. A third agent, such as linezolid, may be added in severe nocardiosis. The duration of treatment should be 6-12 months in immunocompetent individuals and at least 12 months in immunocompromised individuals and those with CNS involvement to prevent relapse [14,15].

## Conclusions

We presented a rare and challenging case of disseminated *Nocardia brasiliensis* infection manifesting as brain and skin abscesses. This case highlights the importance of considering *Nocardia* as a potential pathogen, particularly in patients with a travel history to endemic regions or those with unusual clinical presentations, such as brain abscesses and skin infections. Diagnosing *Nocardia* infection can be challenging due to its slow growth and variable staining properties, necessitating extended culture incubation and molecular techniques for early identification. This case also highlights the limitations of imaging studies in diagnosing *Nocardia* brain abscesses, as the patient’s non-MR conditional pacemaker precluded MRI evaluation and initially led to a diagnostic challenge. Early recognition, appropriate diagnostic approaches, and tailored antimicrobial therapy are crucial in successfully managing disseminated *Nocardia* infections. This case underscores the need for vigilance in considering *Nocardia* as a differential diagnosis, particularly in patients with complex clinical presentations, ensuring timely and effective management to improve patient outcomes.
